# Evaluation of Shifts of Gene Transcription Levels of Unicellular Green Alga *Chlamydomonas reinhardtii* Due to UV-C Irradiation

**DOI:** 10.3390/microorganisms11030633

**Published:** 2023-03-01

**Authors:** Akihito Nakanishi, Nanami Ozawa, Masahiko Watanabe

**Affiliations:** 1 Graduate School of Bionics, Tokyo University of Technology, 1404-1, Katakuramachi, Hachioji, Tokyo 192-0982, Japan; 2 School of Bioscience and Biotechnology, Tokyo University of Technology, 1404-1, Katakuramachi, Hachioji, Tokyo 192-0982, Japan

**Keywords:** *Chlamydomonas reinhardtii*, UV-C irradiation, cell-sterilizing system, quantitative PCR, lipid metabolism

## Abstract

Green algae produce valuable lipids as carbon-recycling resources. Collecting whole cells with the intracellular lipids could be efficient without cell burst; however, direct use of the cells causes microbial contamination in environments. Then, UV-C irradiation was selected to satisfy the requirements of avoiding the cell burst and sterilizing cells with *Chlamydomonas reinhardtii*. UV-C irradiation with 1.209 mW·cm^−2^ showed enough sterilization activity for 1.6 × 10^7^ cells·mL^−1^ of *C. reinhardtii* in a depth of 5 mm for 10 min. The irradiation showed no effects to composition and contents of the intracellular lipids. From the viewpoint of transcriptomic analysis, the irradiation displayed possibilities of (i) inhibition of the synthesis of lipids due to decrement of the transcription of related genes, such as *diacylglycerol acyl transferase* and *cyclopropane fatty acid synthase*, and (ii) activation of lipid degradation and the production of NADH_2_^+^ and FADH_2_ due to increment of the transcription of related genes, such as *isocitrate dehydrogenase*, *dihydrolipoamide dehydrogenase* and *malate dehydrogenase*. Irradiation until cell death could be insufficient to shift the metabolic flows even though the transcriptions were already shifted to lipid degradation and energy production. This paper is the first report of the response of *C. reinhardtii* to UV-C irradiation on the transcription level.

## 1. Introduction

Industrial microorganisms have many excellent qualities, such as proliferative capacity, biosafety and metabolic pathways well-suited to the production of value-added chemicals [1]. Although industrial microorganisms are useful for production, most producing systems using heterotrophic microorganisms have led to suppressed economic costs and avoidance of microorganism contamination due to expensive and nutritious carbon sources (e.g., glucose) [2,3]. On the other hand, a system using several green algae, such as *Chlamydomonas* sp. and *Chlorella* sp., which are superior to terrestrial plants in terms of their lipid and carbohydrate production, with 10~50 times higher assimilation activity of CO_2_ [4,5], can lower the economic cost and escape contamination by using free and poor nutrients as a carbon source. Although green algae prioritize bioproductions, such as those discussed above, the collection processes of intracellular contents should be considered because of their deep relation to the economic cost [6]. In particular, collection of the intracellular contents from the dead cells is generally difficult due to cell burst, which disperses the intracellular contents in response to stress [7,8]. In the extraction step, cost reduction can be expected with easy harvesting of the cells containing the value-added intracellular contents. Thus, it is preferable to collect cells in a viable state for easy collection of their contents. However, to avoid microbial contamination in the environment, the cells must be completely sterilized using several methods such as UV-C irradiation, autoclaving and dry-air sterilization [9]. Despite the conflicting requirements, the cells should be sterilized as quickly as possible after death. Sterilization methods suitable for use in the material production of microorganisms should also possess advantages such as a short processing time, low energy consumption and low capital investment costs. UV-C irradiation has seen extensive use as a strong sterilization tool, and there have been many achievements in the treatment of various microorganisms with these advantageous properties [10], including green alga *C. reinhardtii* [11]. However, during bioproduction, the irradiation effect should be evaluated for not only the cell viability, but also the changes in intracellular contents. For instance, as an effect of irradiation, the lipid compositions change after UV-C irradiation [12]. Additionally, when using intracellular metabolites as the value-added compounds, the shifts of the metabolic flows due to the irradiation should also be estimated. The shifts of the metabolites as targeted compounds with irradiation were detected, resulting in the selection of other sterilization methods instead of irradiation. To date, in the field of bioproduction with green algae, although the responses of metabolic flows toward stresses such as pH and salt have been reported [13,14,15], there have been few reports regarding the intracellular responses toward UV-C irradiation as an academic explanation. One report addressed cell responses to UV-C irradiation; however, the report only explained that the irradiated cells induced apoptosis by repressing the defender against the apoptotic death gene [16,17]. Regarding the responses to UV-C, the effects on the intracellular contents and whole transcriptions relating material production to *Chlamydomonas* sp. have yet to be determined. UV-C irradiation is a beneficial sterilization method [18,19]; thus, revealing the responses of transcriptions relating the metabolic flows to UV-C irradiation would be meaningful in bioproduction using microorganisms [20].

This study aimed to reveal the responses of *C. reinhardtii* as a substance-producing strain to UV-C irradiation in order to maintain the status of lipids, which are valuable intracellular contents. Changes in lipid contents and compositions are undesirable for material production under a sterilizing treatment, meaning that a treatment such as UV-C irradiation should not affect lipid decomposition except for sterilization. Assuming the lipid extraction from cells, this study confirmed whether the UV-C irradiation could affect lipids as intracellular contents because the details of the cellular response to irradiation were unknown. Then, regarding the effects of UV-C irradiation on the cells, the changes in viability and the lipid leakage from the cells, the lipid contents and compositions were evaluated to evaluate the effect of the UV-C irradiation. Furthermore, the gene transcriptions were quantified with quantitative PCR to estimate the changes in intracellular metabolic flow caused by the irradiation. This study suggested that UV-C irradiation to *C. reinhardtii* would be useful for sterilization of cells, obtaining lipids as bioproducts.

## 2. Materials and Methods

### 2.1. Microalgal Strain and Culturing Condition

*Chlamydomonas reinhardtii* strain C-9: NIES-2235 was purchased from the National Institute for Environmental Studies (NIES) (Tsukuba, Ibaraki, Japan). *C. reinhardtii* was cultivated in a photobioreactor (PBR) filled with Modified Bold (MB) medium, as described in a previous study [15]. The culture condition was controlled as described below. Volume: 100 mL in a glass vessel; light intensity: 100 µmol photons·m^−2^·s^−1^ with white fluorescent lamps; gas bubbling: 0.8% CO_2_ gas at 0.2 vvm; temperature: room temperature (23 °C). Cell growth was evaluated based on values of optical density (OD) at 750 nm with a spectro-photometer U-2900 (Hitachi, Tokyo, Japan) via appropriate calibration curves for OD_750_ versus dry cell weight (DCW) (mg∙mL^−1^) and cell numbers (cells∙mL^−1^), respectively. To control the cells, the pH of the broth was measured with the pH meter FEP20 (Mettler Toledo, Tokyo, Japan). Nitrate concentration was measured using an optical method: the broth was centrifuged at 5000× *g* for 1 min at 23 °C, and the supernatant was filtered with a 0.45 μm filter (Millex^Ⓡ^-LCR 13 mm, Millipore, Carrigtwohill, Ireland); the flow through was diluted 50-fold with distilled water; the absorbance of the diluted supernatant was measured at 220 nm (i.e., Abs_220_) [21] using a spectro-photometer U-2900 (Hitachi High-Technologies Corporation, Tokyo, Japan). The residual nitrate content was detected with an appropriate calibration curve for the value of Abs_220_ versus nitrate concentration. 

### 2.2. Cell Sterilization with UV-C Irradiation

Cells were collected and adjusted to 0.60~4.0 mg DCW (0.93~6.2 × 10^7^ cells) in 2.0 mL of phosphate-buffered saline (PBS) (pH = 7.4) on a glass Petri dish of 33 mm diameter. The depth of the mixture containing the cells was 5.0 mm. The cell density was evaluated using a calibration curve of DCW versus OD_750_. The prepared cells were irradiated with a UV irradiator Etbella (SEVEN BEAUTY Co., Ltd., Tokyo, Japan) equipped with a UV-C lamp (with the ultraviolet radiation at the length of 9.0 cm: 1.209 mW·cm^−2^). The cell viability was evaluated using the neutral red method described in a previous study [22]. The UV-C irradiation-induced decrements in the cell viabilities were evaluated, using each viability at 0 min as a control. The changes in the cell densities caused by the UV-C irradiation were also evaluated, with each cell density at 0 min as a control.

### 2.3. Evaluation of Lipid Composition and Quantity in Cells with Gas Chromatography

Cells were broken with 0.5 mm glass beads, and then total extracted lipids were methyl esterified with a fatty acid methylation kit (Nacalai Tesque, Kyoto, Japan). The fatty acid methyl esters were identified and quantified with a capillary gas chromatograph GC-2025 (Shimadzu, Kyoto, Japan) equipped with a DB-23 capillary column (60 m, 0.25 mm internal diameter, 0.15 μm film thickness) (Agilent Technologies, Santa Clara, CA, USA) followed by the previous method [15]. Heptadecanoic acid (Sigma-Aldrich Co., St. Louis, MO, USA) was used as an internal standard; rapeseed oil (Merck KGaA, Darmstadt, Germany) was used as a quantitative standard. The changes in the contents and compositions of lipids induced by the UV-C irradiation were also evaluated, with those at 0 min used as a control.

### 2.4. Measurement of Levels of Gene Translation

*C. reinhardtii* was cultivated in MB medium under PBR conditions, as mentioned above. First, approximately 5 mg of cells was collected, measured with the calibration curve of OD_750_ dry cell weight via centrifugation at 21,500× *g* for 5 min. The collected strains were mixed with 50 µL of QIAzol Lysis Reagent (QIAGEN, Tokyo, Japan) and shaken for 5 min. After keeping the shaken samples at 23 °C for 5 min, 10 µL of chloroform was added and placed on ice for 3 min. The treated samples were centrifuged at 21,500× *g* for 15 min; the supernatant was shaken with 25 µL of isopropanol; the mixture was placed at 23 °C for 10 min. The supernatant was discarded after centrifugation at 21,500× *g* for 10 min, and the precipitant was rinsed with 1 mL of 70% ethanol. The rinsed sample was dried with lyophilizer Refrigerated CentriVap Benchtop Vacuum Concentrator (Labconco, MO, USA), and the dried precipitant was dissolved in 10 µL of RNase free water. The prepared sample as total RNA was used to synthesize complementary DNA (cDNA) using a ReverTra Ace qPCR RT Master Mix with a gDNA Remover (TOYOBO, Osaka, Japan). With the cDNA, quantitative PCR was performed with THUNDERBIRD SYBR qPCR Mix (TOYOBO) using Mx qPCR Systems (Agilent, CA, USA). The average threshold cycle values were evaluated throughout the logarithmic amplification phase and were normalized by the level of the *ATPase*. The quantitative PCR primers (Supplemental Tables S1~S5) were designed based on the Primer3Plus algorithm (https://dev.primer3plus.com/index.html, accessed on 7 April 2021), using information from each predicted gene sequence obtained via the genome information of NCBI. The changes in the transcription levels as a result of the UV-C irradiation were also evaluated, with those at 0 min used as a control.

## 3. Results and Discussion 

### 3.1. Effect of UV-C Irradiation on Cell Viability

**Figure 1:**
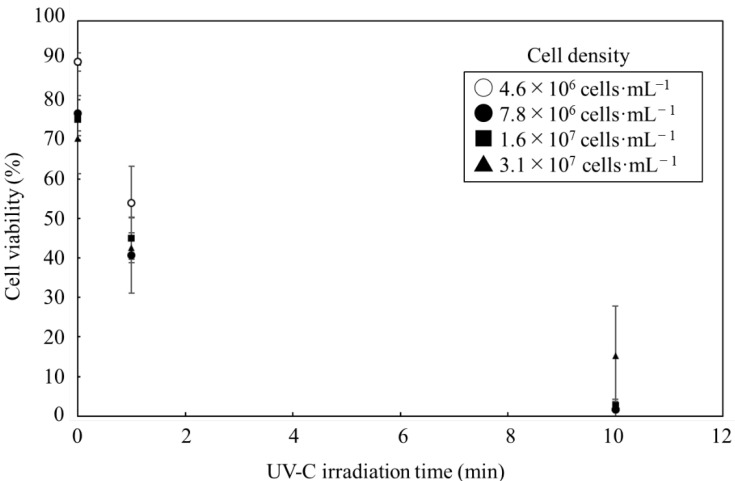
Effect of UV-C irradiation for cell sterilization. Cell viability of *C. reinhardtii* was evaluated in each cell density after UV-C irradiation. Cells were irradiated with a UV irradiator (1.209 mW·cm^−2^) in 5.0 mm depth of 2.0 mL of PBS on a 33 mm glass petri dish. The cell densities were shown as cycle 4.6 × 10^6^ cells·mL^−1^, diamond 7.8 × 10^6^ cells·mL^−1^, square 1.6 × 10^7^ cells·mL^−1^ and triangle 3.1 × 10^7^ cells·mL^−1^. Error bars indicate the standard deviation (SD) of three replicate experiments (n = 3).

In order to achieve efficient sterilization of *C. reinhardtii* cells in this study, the UV-C irradiation was examined under each condition (Figure 1). The examinations revealed that the cell viability was negatively correlated with the duration of the irradiation with the cell densities of 4.6 × 10^6^~3.1 × 10^7^ cells·mL^−1^ (0.30~2.0 mg·mL^−1^) at a depth of 5.0 mm for 10 min. In cell densities of 1.6 × 10^7^ cells·mL^−1^ and less, although only 40 to 55% of the cells were sterilized in 1 min using UV-C irradiation, most of the cells were completely sterilized in 10 min. At 3.1 × 10^7^ cells·mL^−1^, the cells were sterilized, correlating to the length of the UV-C irradiation; however, almost 15% of cells were alive even when UV-C irradiation was implemented for 10 min. 

The incomplete cell death at 10 min irradiation was detected in the cell density condition as 3.1 × 10^7^ cells·mL^−1^. Masuda et al. reported that increasing the cell density was associated with a decrease in the light intensity, even at the same depth [23]. Survival following a 10 min irradiation was strongly related to the cell density. The UV-C irradiation in this study was performed with 1.209 mW·cm^−2^ for 10 min; the total energy was 725.4 mJ·cm^−2^. The condition was optimized to sterilize most of the cells efficiently when the cell density was controlled at 1.6 × 10^7^ cells·mL^−1^. Sharma et al. reported that 17% of *Tetraselmis* sp. at 1.0 × 10^6^ cells·mL^−1^ were alive after the UV-C irradiation of 1000 mJ·cm^−2^ [12]. The irradiation in this study was more efficient than in previous experiments because the survival ratio was almost 0%, even at 16 times higher cell density with 27% lower total energy. The UV-C irradiation could induce the apoptosis of *C. reinhardtii* [17], downregulating a homologue of the defender for an apoptosis-inducing gene such as *dad1* [16]. The irradiation could not cause cell death by directly destroying the intracellular contents, but it could induce the programed death, potentially causing minor damage to intracellular contents such as lipids. However, cell death induced by UV-C irradiation could possibly cause cell burst; additionally, the UV light might change triplet oxygen into highly reactive single oxygen ^1^O_2_ or promote the oxidation of polyunsaturated fatty acids (PUFAs). Oxidation generates hydrogen peroxides and secondary oxidation products affecting compounds such as triacylglycerols [24,25]. Therefore, the effects of the UV-C irradiation should be evaluated based on intracellular contents.

### 3.2. Effect of UV-C Irradiation on Cell Structure

**Figure 2:**
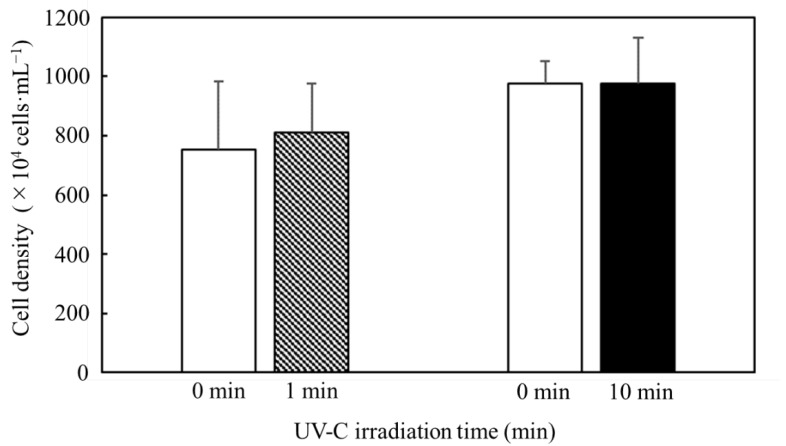
Effect of UV-C irradiation for cell burst. Cell densities before/after UV-C irradiation were quantified with a hemocytometer. The prepared cell densities were 4.8 × 10^6^~1.2 × 10^7^ cells·mL^−1^, and the cells were treated by UV-C irradiation. Error bars indicate the SD of three replicate experiments (n = 3).

Harvesting the cells while maintaining their intracellular content was related to the easy collection of value-added metabolites because after the dispersion by the cell burst in the media, the metabolites were difficult to collect. Therefore, although the *C. reinhardtii* cells had to be sterilized with the UV-C irradiation to avoid microbial contamination, the sterilized cells easily maintained the cell structures for collection of the intracellular contents. Then, in order to confirm whether UV-C irradiation causes not only cell death, but also cell burst, the UV-C irradiation was implemented to confirm the effect on the cell density under cell sterilization conditions adjusted to 7.8 × 10^6^ cells·mL^−1^ at a depth of 5 mm in 10 min (Figure 2). The shifts in the cell densities after 1 min and 10 min of UV-C irradiation were evaluated with a t-test; the null hypothesis of the shifts was not rejected despite the high significance levels (*p* = 0.483 and *p* = 0.997, respectively).

The cells often showed bursts, such as apoptosis and necrosis, due to stresses because these cannot maintain the cell wall [7], resulting in the cell density decreasing after the cell burst. Then, in this study, the effect of the UV-C irradiation confirmed whether the irradiation could trigger the cell burst through evaluation of decreasing cell densities (Figure 2). According to the results relating to the cell density in Figure 1 and Figure 2, the UV-C irradiation failed to significantly decrease the cell densities, even though most cells were sterilized by the irradiation for 10 min. Therefore, it was possible to maintain the intracellular contents even with UV-C irradiation so that intracellular contents such as lipids could be easily collected with the cells.

### 3.3. Effect of UV-C Irradiation on Lipids

**Figure 3:**
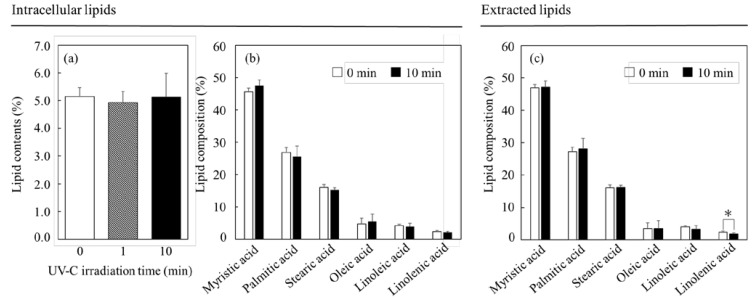
Effects of UV-C irradiation on lipids. The cells at 7.8 × 10^6^ cells·mL^−1^ were treated with UV-C irradiation in this study. Lipid contents and compositions in the cells are shown in (**a**,**b**). In (**a**), showing the lipid contents of the cells, the bars of white, gray and black show the contents at 0 min, 1 min and 10 min of UV-C irradiation, respectively (n = 3). In (**b**), showing the lipid compositions of the cells, the bars of white and black show the compositions at 0 min and 10 min of UV-C irradiation (n = 3). On the other hand, the lipids extracted from the 1.6 × 10^7^ cells were treated with the same level of UV-C irradiation. In (**c**), the bars of white and black show the compositions at 0 min and 10 min of UV-C irradiation (n = 3). The symbol * meant *p* < 0.05.

The shifts in the contents and compositions of the lipids, some of the useful intracellular substances, were analyzed after the UV-C irradiation (Figure 3). The results of the analysis are shown in Figure 3a. The lipid contents were 5.2 ± 0.3%, 4.9 ± 0.4% and 5.1 ± 0.9% with 0 min, 1 min and 10 min irradiations, respectively. Therefore, 10 min irradiation clearly showed few changes in the intracellular lipid contents with no significant differences. In addition, the composition of the intracellular lipids also showed few differences, even in relation to UV-C irradiation (Figure 3b); the composition of the extracted lipids also displayed few changes with no significant differences, excluding a case of linolenic acid (Figure 3c). The change in linolenic acid was indicated by the significance level of *p* = 0.0235 (* *p* < 0.05), which indicated that part of the extracted lipids was affected by the UV-C irradiation. 

The shifts in the contents and the compositions of the lipids were evaluated after 10 min of the lethal UV-C irradiation (Figure 3). Although the UV-C irradiation affected the composition of the extracted lipids with the trend of decreasing the ratio of linolenic acid, the irradiation did not influence the composition of the intracellular lipids. Additionally, the irradiation did not affect the intracellular lipid contents. The results indicated that the cell structures and the components may have protected the intracellular lipids, thus avoiding lipid degradation. The results revealed that the UV-C irradiation as performed under the conditions outlined in this study could affect only the sterilization but not the lipids, depending on a lack of significant difference, indicating that irradiation could be a useful tool for the use of intracellular lipids of *C. reinhardtii*. It cannot be denied that the intracellular lipids showed few effects in response to the irradiation, but the gene transcription relating deeply to the metabolic flows showed huge influences. 

### 3.4. Selection of a Housekeeping Gene for Quantitative PCR under UV-C Irradiation

**Figure 4:**
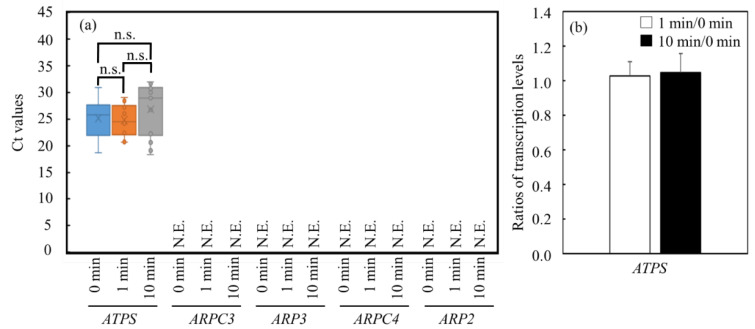
Ratios of transcription levels of estimated housekeeping genes in *C. reinhardtii* C-9: NIES-2235 after UV-C irradiation. (**a**) Boxplot representation of raw Ct values obtained from amplification curves (n = 3). Lower and upper boundaries of the box indicate the 25th and the 75th percentile, the thin line within the box marks the median. The Ct values were evaluated at 0 min, 1 min and 10 min of UV-C irradiation. (**b**) Ratios of transcription levels of nominated *ATPS* before/after 10 min UV-C irradiation were evaluated with quantitative PCR. Error bars indicate the SD of three replicate experiments (n = 3). Not evaluated was abbreviated as N.E. Housekeeping genes, *ATPS*: ATPase beta chain of ATP synthase gene; *ARPC3*: actin-related protein Arp2/3 complex, subunit ARPC3 gene; *ARP3*: actin-related protein Arp2/3 complex, subunit Arp3 gene; *ARPC4*: actin-related protein Arp2/3 complex, subunit ARPC4 gene; *ARP2*: actin-related protein Arp2/3 complex, subunit Arp2 gene.

Quantitative PCR was performed to clarify how the cells attempted to metabolically respond to the shifts in the gene transcriptions, using the UV-C irradiation as the source of stress. In this study, five housekeeping genes were suggested for the quantitative PCR as follows, according to previous quantitative PCR conditions: *ATPS* (ATPase beta chain of ATP synthase gene) [26], *ARPC3* (actin-related protein Arp2/3 complex, subunit ARPC3 gene) [27], *ARP3* (actin-related protein Arp2/3 complex, subunit Arp3 gene) [27], *ARPC4* (actin-related protein Arp2/3 complex, subunit ARPC4 gene) [27] and *ARP2* (actin-related protein Arp2/3 complex, subunit Arp2 gene) [27]. As the results show, although few transcriptions of *ARPC3*, *ARP3*, *ARPC4* and *ARP2* were detected, that of *ATPS* could be stably detected with/without the UV-C irradiation (Figure 4). 

The effects on the cells from the UV-C irradiation were evaluated with quantitative PCR because the cells would response on the transcriptional levels toward the stress soon. *ATPS* was stably detected according to the results of the quantitative PCR (Figure 4). The *ATPS*, the gene codes part of the ATP synthetase [26], should be constantly expressed in the cells, even with the UV-C irradiation, so that the gene transcription is stably detected via the quantitative PCR. Interestingly, *ATPS* was constantly detected, even after the UV-C irradiation. On the other hand, the gene transcriptions relating to β-actin were not stably detected, indicating that those genes could be drastically affected by the irradiation. The constant transcription of the housekeeping gene would be required for quantitative PCR, even after the irradiation, so *ATPS* was selected as the housekeeping gene depending on the constant detection of the genes in this study.

### 3.5. Analysis of Transcription-Level under UV-C Irradiation

**Figure 5:**
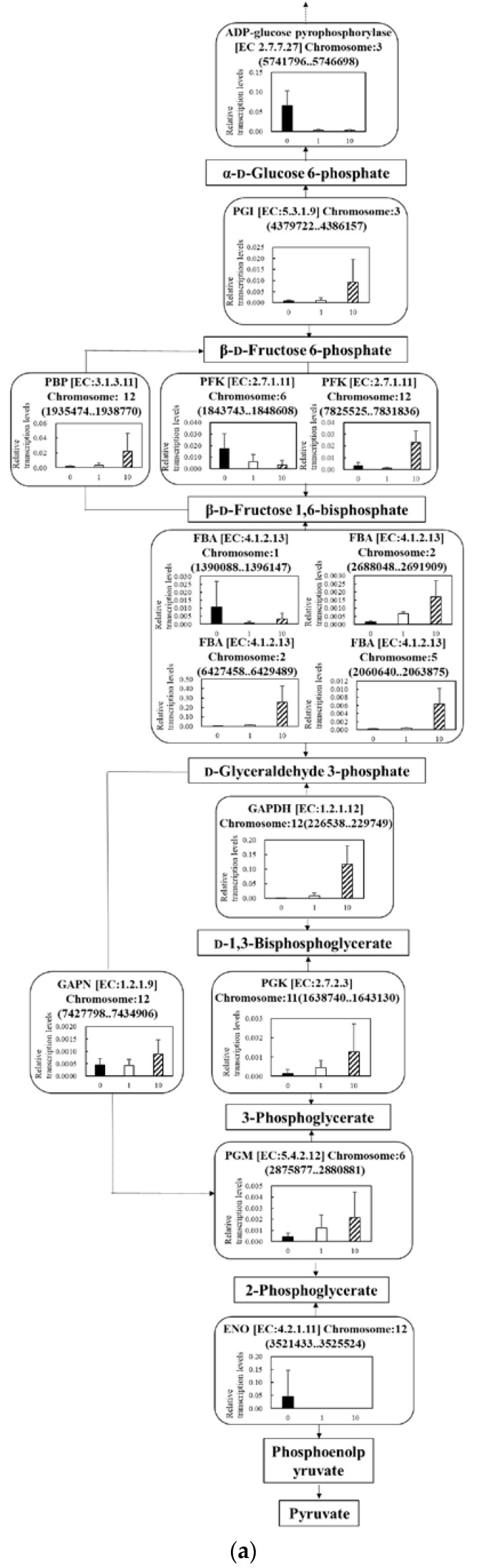

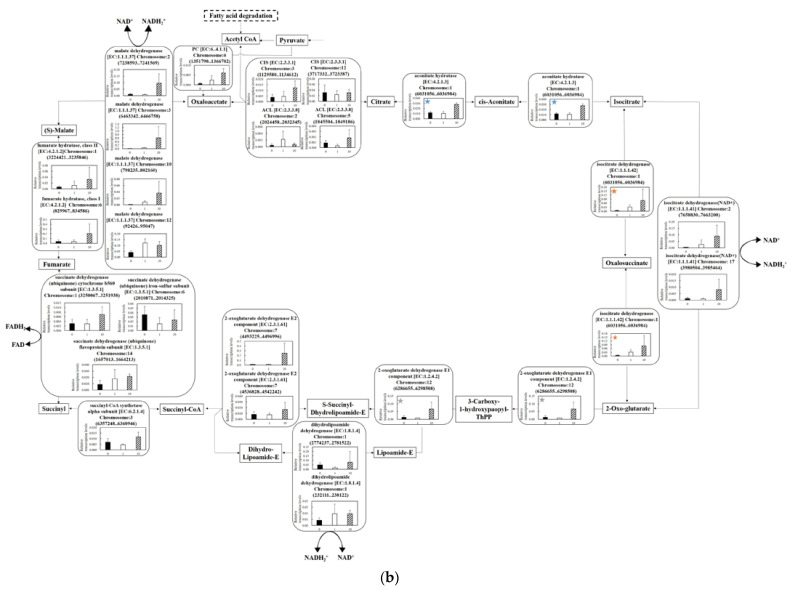

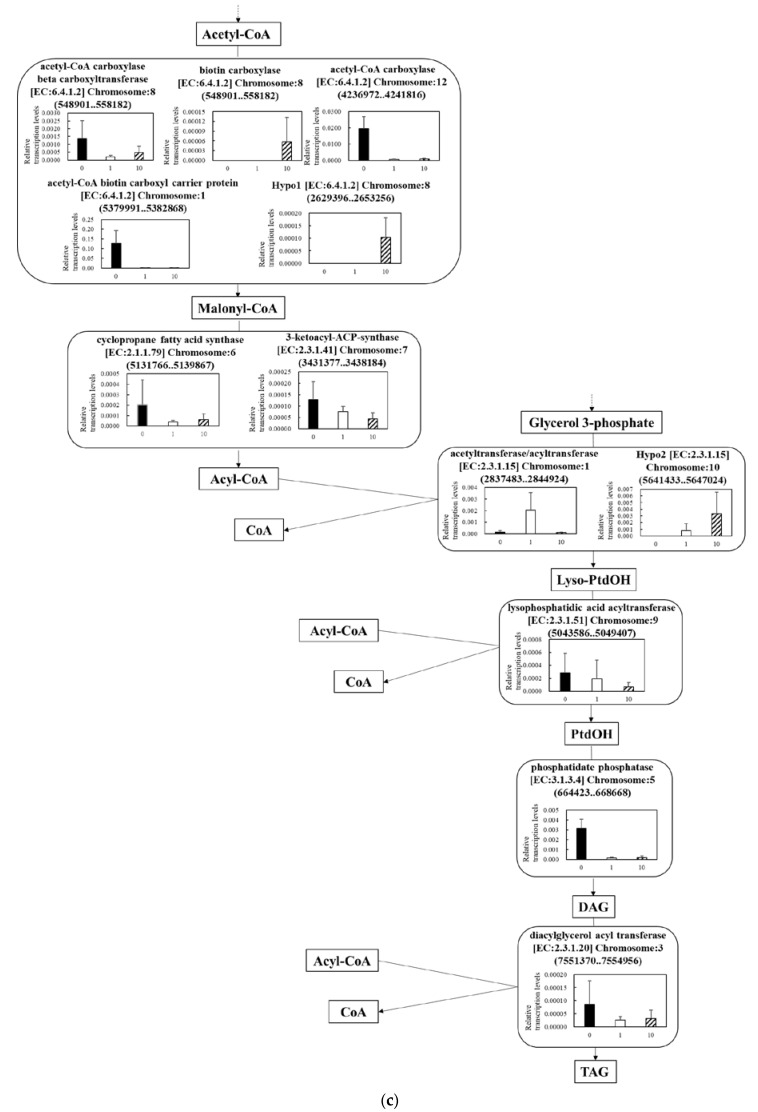

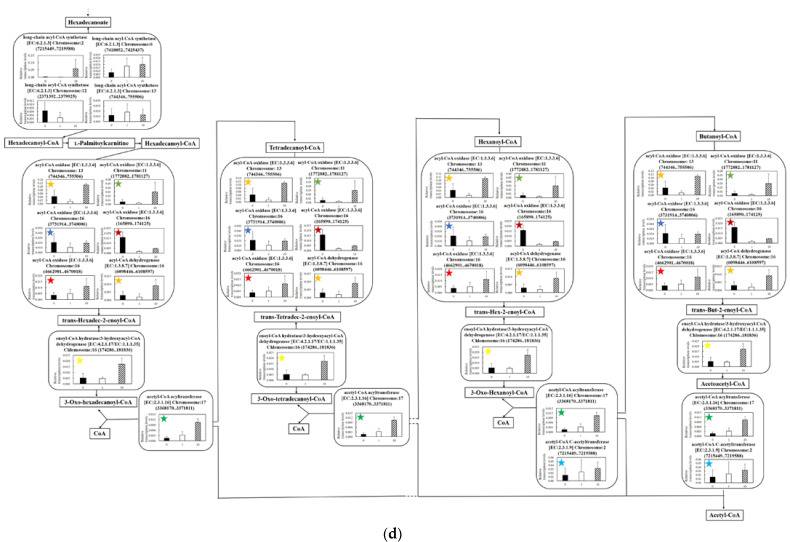
Relative quantification of mRNA of *C. reinhardtii* at 0 min, 1 min and 10 min of UV-C irradiation: (**a**) glycolysis; (**b**) TCA cycle; (**c**) lipid-synthetic pathway; (**d**) lipid-degrading pathway. Data were shown as relative mRNA transcription levels normalized by the level of *ATPS* as a housekeeping gene. Relative transcription levels at 0 min, 1 min and 10 min of UV-C irradiation time were shown in black, white and striped, respectively. Error bars indicate SD of 3~6-time replicates experiments. Metabolites, glycerol 3-phosphate: G3P; lysophosphatidic acid: Lyso-PtdOH. Enzymes, hypo-ligases: Hypo1; hypo-transferases: Hypo2; phosphoglucoisomerase: PGI; fructose-1,6-bisphosphatase: FBP; phosphofructokinase: PFK; fructose-bisphosphate aldolase: FBA; glyceraldehyde 3-phosphate dehydrogenase: GAPDH; glyceraldehyde-3-phosphate dehydrogenase: GAPN; phosphoglycerate kinase: PGK; phosphoglycerate mutase: PGM; enolase: ENO; pyruvate carboxylase: PC; citrate synthase: CIS; ATP citrate (pro-S)-lyase: ACL. The same-colored stars in (**b**,**d**) indicated the same genes.

While cells undergo rapid death due to UV-C irradiation, these cells were forced to adapt to face the stress that leads to fatality. Since the cells were required to modify their metabolic flows quickly to survive lethal stress [28], the mRNA transcription levels demonstrated a rapid response. Then, the intracellular response to the UV-C irradiation was evaluated with quantitative PCR (Figure 5). In particular, several important pathways and cycles, such as (a) central metabolism, (b) the TCA cycle deeply relating to energy production, (c) lipid production and (d) lipid degradation, were evaluated via quantitative PCR. When the cell concentration was adjusted at 1.6 × 10^7^ cells·mL^−1^, the cell viability exponentially decreased due to the UV-C irradiation, resulting in 75.0 ± 13.7%, 45.0 ± 5.3% and 2.7 ± 1.5% at 0 min, 1 min and 10 min (Figure 1). The results showed that 40~55% of the cells were sterilized by UV-C irradiation in 1 min, and almost 100% were killed in 10 min. Therefore, when performing the quantitative PCR to analyze the responses of cells as a transcriptome, the time of the UV-C irradiation was set at 1 min as a mid-point and 10 min as the final point.

#### 3.5.1. Effect of UV-C Irradiation on Transcription on Glycolysis

The shift in the transcription levels relating to the central metabolism were analyzed under UV-C irradiation (Figure 5a). The transcription levels of *phosphoglucoisomerase* (*PGI*) [EC:5.3.1.9] Chromosome:3 (4379722..4386157), *fructose-1,6-bisphosphatase* (*FBP*) [EC:3.1.3.11] Chromosome:12 (1935474..1938770), *glyceraldehyde 3-phosphate dehydrogenase* (*GAPDH*) [EC:1.2.1.12] Chromosome:12 (226538..229749), *phosphoglycerate kinase (PGK*) [EC:2.7.2.3] Chromosome:11 (1638740..1643130), *glyceraldehyde-3-phosphate dehydrogenase* (*GAPN*) [EC:1.2.1.9] Chromosome:12 (7427798..7434906), *phosphoglycerate mutase* (*PGM*) [EC:5.4.2.12] and Chromosome:6 (2875877..2880881), which are uniquely enzymatic genes in the central metabolism, were increased by the UV-C irradiation. On the other hand, the transcription level of the gene of enolase (ENO) [EC:4.2.1.11] Chromosome:6 (2875877..2880881), which is also a uniquely enzymatic gene, was drastically depressed by the UV-C irradiation. Phosphofructokinase (PFK) and fructose-bisphosphate aldolase (FBA) have isozymes, and the transcription levels of isozymes were reversed after the UV-C irradiation. In detail, regarding *PFK* [EC:2.7.1.11], the transcription level of *PFK* [EC:2.7.1.11] Chromosome:6 (1843743..1848608) was decreased and that of *PFK* [EC:2.7.1.11] Chromosome:12 (7825525..7831836) was controversially increased; about *FBA* [EC:4.1.2.13], that of *FBA* [EC:4.1.2.13] Chromosome:1 (1390088..1396147) was decreased and those of *FBA* [EC:4.1.2.13] Chromosome:5 (2060640..2063875), *FBA* [EC:4.1.2.13] Chromosome:2 (6427458..6429489) and *FBA* [EC:4.1.2.13] Chromosome:2 (2688048..2691909) increased. 

During carbon fixation in photosynthesis, the carbon source is assimilated into glycolysis after converting CO_2_ into 3-phosphoglyceric acid [29,30], so 3-phosphoglyceric acid is mainly used as a starting compound as the carbon source in glycolysis. This occurs via photosynthesis. *Chlamydomonas* sp. tends to accumulate a carbohydrate as the carbon source in the cell when the cell detects strong stresses, such as nitrogen depletion, in the photosynthesis [29]. In fact, the carbohydrate accumulated in the cells is biosynthesized with ADP-glucose converted via the process of transforming from glucose-6 phosphate to glucose-1 phosphate [29,30]. According to the analytical results for the shifts of the transcription levels from 3-phosphoglycelic acid to glucose-6 phosphate due to the UV-C irradiation, although the levels of *FBA* [EC:4.1.2.13] Chromosome:1 (1390088..1396147) were decreased, those of the other *FBAs* were increased (Figure 5a). The decreased transcription of *FBA* [EC:4.1.2.13] Chromosome:1 (1390088..1396147) was supported by the other *FBA*, and the total transcription of *FBA* was enhanced. Therefore, there was a possibility that *C. reinhardtii* upregulated the biosynthesis pathway of the carbohydrate from 3-phosphoglyceric acid due to the UV-C irradiation and the associated biological response, such as the reactions toward stresses. On the other hand, the carbon sources were converted from 3-phosphoglyceric acid into the TCA cycle via phosphoenolpyruvic acid [30]. The transcription levels of *ENO* [EC:4.2.1.11] Chromosome:6 (2875877..2880881) were depressed by the UV-C irradiation, indicating that *C. reinhardtii* tended not to result in a direct metabolic flow from CO_2_ as a carbon source to the TCA cycle. The summary of those results and discussion displayed the possibility that *C. reinhardtii* actively converted CO_2_ to the carbohydrate as a stock responding to the UV-C irradiation.

#### 3.5.2. Effect of UV-C Irradiation on Transcription on the TCA Cycle

The transcription levels of the genes relating to the TCA cycle were evaluated when the cells were irradiated by the UV-C (Figure 5b). (i) The transcription levels of *citrate synthase* (*CIS*) and *ATP citrate (pro-S)-lyase* (*ACL*), the two types of enzymes relating to the reaction from oxaloacetic acid to citric acid, were affected by the UV-C irradiation. As a result, the transcription levels of *CIS* [EC:2.3.3.1] Chlomosome:3 (1129580..1134612) and *ACL* [EC:2.3.3.8] Chlomosome:5 (1845504..1849186) indicated the max values at 10 min of UV-C irradiation. (ii) For the transcription levels of *pyruvate carboxylase* (*PC*) [EC:6.4.1.1] Chlomosome:6 (1351790..1366702) and *aconitate hydratase* [EC:4.2.1.3] Chlomosome:1 (6031056..6036984), those genes were unique in the related pathway and were increased by the UV-C irradiation. (iii) The oxoglutarate dehydrogenase complexes connecting the metabolic pathway from 2-oxo-glutarate to succinyl-CoA are composed of three units of E1~E3. The E1 is an oxoglutarate dehydrogenase with thiamine pyrophosphate (TPP) as a cofactor; the E2 is dihydrolipoyl succinyltransferase with lipoic acid and coenzyme A as a cofactor; the E3 is dihydrolipoyl dehydrogenase with FAD and NAD as a cofactor [31]. In this condition, these transcription levels tended to increase after UV-C irradiation. (iv) Fumarate hydratase and malate dehydrogenase have two and four isozymes, and all isozymes were enhanced by the UV-C irradiation. In detail, those of *fumarate hydratase* class I [EC:4.2.1.2] Chlomosome:6 (829967..834586), *fumarate hydratase* class II [EC:4.2.1.2] Chlomosome:1 (3224421..3235846), *malate dehydrogenase* [EC:1.1.1.37] Chlomosome:2 (7238593..7241569), *malate dehydrogenase* [EC:1.1.1.37] Chlomosome:3 (6463342..6466758) and *malate dehydrogenase* [EC:1.1.1.37] Chlomosome:10 (798235..802160) showed the highest values for the 10 min irradiation; *malate dehydrogenase* [EC:1.1.1.37] Chlomosome:12 (92426..95047) also showed the max value for 1 min irradiation.

The transcription levels in the TCA cycle should be analyzed because the cycle was important to the production of the biological energy relating to survival under stress conditions (Figure 5b). Acetyl-CoA is theoretically produced by decomposing the lipids, and the transcription levels of the genes relating to the reactions of the lipid degradation were activated, as discussed below (Figure 5d). (i) In the TCA cycle, the transcription levels of *citrate synthase* and *ATP citrate (pro-S)-lyase* were maintained and activated after the UV-C irradiation, indicating the possibility that the acetyl-CoA could efficiently be introduced into the TCA cycle. (ii) Regarding the increment of the transcription level of *PC*, although the aim was not to show the enhancement required to produce pyruvate according to the transcription analysis on glycolysis (Figure 5a), the cells tended to supply oxaloacetate, which was simultaneously required with the introduction of acetyl-CoA, from pyruvate, even though the production was low. (iii) The transcription levels of *oxoglutarate dehydrogenase complex* controlling the metabolic reactions from 2-oxo-glutarate and succinyl-CoA increased as the response and could possibly perform enhanced production of NADH_2_^+^. (iv) Fumarate hydratase and malate dehydrogenase have two and four isozymes, respectively, and their transcription levels were increased due to the irradiation. The gene cluster composed of those genes was required for reaction at the starting point in the TCA cycle so that the gene transcriptions in the cluster possibly upregulated the pathway for producing energy. In particular, the enhanced transcription levels of *malate dehydrogenase* were important since the enzymes could produce NADH_2_^+^. 

Regarding the production of biological energy, the preparation for the electron transport chain is very important for obtaining the reduction power. The transcription levels of all enzyme genes relating to the production of NADH_2_^+^ from NAD^+^ (*isocitrate dehydrogenase*: the reaction from isocitrate to 2-oxo-glutarate; *dihydrolipoyl dehydrogenase*: the reactions from lipoamide-E to dihydro-lipoamide-E; *malate dehydrogenase*: the reactions from malate to oxaloacetate) increased due to the UV-C irradiation. The produced NADH was used to generate the proton gradient for the electron transport chain in the mitochondrial inner membrane, resulting in ATP production as an oxidative phosphorylation [32]. Additionally, the transcription levels of most genes relating to the TCA cycle displayed maintenance or enhancement; in the event of a decrease in either, other isozyme genes complemented the depressed genes. Therefore, the results indicated that the cells attempted to activate the reaction in the TCA cycle to generate energy as a response to the UV-C irradiation. Using the lipid as the carbon source, *C. reinhardtii* cells could obtain this energy, generating NADH_2_^+^ by introducing acetyl-CoA into the TCA cycle due to the UV-C irradiation. However, this finding contrasted with the knowledge that *C. reinhardtii* tended to use the metabolic flow with CO_2_ as the carbon source for carbohydrate production, providing the nutrient carbon stock.

#### 3.5.3. Effect of UV-C Irradiation on Transcription on Lipid Synthesis

The shifts of the transcription levels in the metabolic pathway from acetyl-CoA to Triacylglycerol (TAG) as a lipid production pathway due to the irradiation were analyzed (Figure 5c). (v) Regarding the five isozymes connecting the metabolic pathway from acetyl-CoA to malonyl-CoA, although the transcription levels of *acetyl-CoA carboxylase beta carboxyltransferase* [EC:6.4.1.2] Chromosome:8 (548901..558182), *acetyl-CoA carboxylase* [EC:6.4.1.2] Chromosome:12 (4236972..4241816) and *acetyl-CoA biotin carboxyl carrier protein* [EC:6.4.1.2] Chromosome:1 (5379991..5382868) decreased in response to the UV-C irradiation, those of *biotin carboxylase* [EC:6.4.1.2] Chromosome:8 (548901..558182) and *hypo-ligases* (*Hypo1*) [EC:6.4.1.2] Chromosome:8 (2629396..2653256) increased, indicating the switching of the transcriptional genes according to the UV-C stress. (vi) Those of *cyclopropane fatty acid synthase* [EC:2.1.1.79] Chromosome:6 (5131766..5139867) and *3-ketoacyl-ACP-synthase* [EC:2.3.1.41] Chromosome:7 (3431377..3438184) relating to the metabolic reaction from malonyl-CoA to acyl-CoA were depressed by the irradiation. (vii) The transcription levels were also switched in the pathway relating the reactions from glycerol 3-phosphate (G3P) to lysophosphatidic acid (Lyso-PtdOH). That of *acetyltransferase/acyltransferase* [EC:2.3.1.15] Chromosome:1 (2837483..2844924) temporally increased at 1 min irradiation and decreased at 10 min; that of *hypo-transferases* (*Hypo2*) [EC:2.3.1.15] Chromosome:10 (5641433..5647024) was enhanced at 10 min. (viii) All transcription levels relating the reactions from Lyso-PtdOH to TAG were depressed by the irradiation.

Green algae generally accumulate lipids in response to stresses, such as a heavy shift in pH and/or salinity, and there were several reports regarding the transcription levels in the lipid synthesis under stress conditions [13,15]. For instance, the increment of the transcription levels of the genes relating to the lipid synthesis, such as *ß-carboxyltransferase* (*BXC1*) and *acyl-ACP thiolase* (*FAT1*), was revealed under the alkalic broth condition [13]; an increase in the transcription of genes, such as *pyruvate ferredoxin oxidoreductase* (*PFOR*) and *pyruvate decarboxylase* (*PDC*), was shown under the salinity stress [15]. However, there has been no report regarding UV-C irradiation as the stress, so the report in this study may have not only industrial but also academic value. This study revealed that the transcription levels of the genes in the lipid synthesis system were affected by the UV-C irradiation. In particular, the effects were significant with regard to the decreasing transcription levels in the reactions from malonyl-CoA to acyl-CoA, shown as (vi), and in those from Lyso-PtdOH to TAG, shown as (viii). As demonstrated by the results of (vi), a decrease in the production of acyl-CoA as a resource of TGA was implied, meaning that the biosynthesis pathway from lyso-PtdOH to TAG could be downregulated. On the other hand, according to the results of (viii), the depression of the biosynthesis of TAG from lyso-PtdOH was also implied. Therefore, these results strongly indicate the possibility that *C. reinhardtii* responded to the lethal UV-C irradiation by suppressing the biosynthesis of TAG. As shown below, the transcription levels of the enzyme genes relating to producing acetyl-CoA in the lipid degradation pathway were enhanced (Figure 5d) so that the decrement of those relating to the lipid synthesis could be meaningful as a response to UV-C irradiation (Figure 5c). 

#### 3.5.4. Effect of UV-C Irradiation on Transcription of Lipid Degradation

The shifts in the transcription levels of the metabolic pathway from hexadecanoate to acetyl-CoA as a lipid degradation pathway due to the irradiation were analyzed (Figure 5d). (ix) Long-chain acyl-CoA synthetase reacting hexadecanoate to hexadecanoyl-CoA has isozymes [33]. Regarding the enzyme genes, the transcription level of *long-chain acyl-CoA synthetase* [EC:6.2.1.3] Chlomosome:2 (7215449..7219588) was increased by the irradiation and *long-chain acyl-CoA synthetase* [EC:6.2.1.3] Chlomosome:12 (2371392..2379925) was decreased, estimating the switching of the enzymes. (x) Acyl-CoA oxidase relating to the reactions from hexadecanoyl-CoA to trans-Hexadec-2-enoyl-CoA has isozymes [34]. Regarding those genes, although the transcription level of acyl-CoA oxidase [EC:1.3.3.6] Chlomosome:16 (165890..174125) decreased as a result of the UV-C irradiation, those of other isozymes maintained or increased. (xi) Regarding the genes of the enzymes in the metabolic flows from trans-Hexadec-2-enoyl-CoA to 3-Hydroxyhexadecanoyl-CoA, *enoyl-CoA hydratase/3-hydroxyacyl-CoA dehydrogenase* [EC:4.2.1.17/EC:1.1.1.35] Chlomosome:16 (174286..181836) was enhanced by the irradiation. (xii) The transcription levels of *acetyl-CoA acyltransferase* [EC:2.3.1.16] Chlomosome:17 (3368170..3371811) and *acetyl-CoA C-acetyltransferase* [EC:2.3.1.9] Chlomosome:2 (7215449..7219588) as the genes related to producing acetyl-CoA were increased by the irradiation. 

Thus far, there have been few reports on the transcription levels relating to lipid degradation under the stress of UV-C irradiation. The same is true of studies on lipid synthesis, illustrating the academic importance of this research. Green algae generally accumulate lipid in the cells as a response to stress [35]. Thus, the results of transcriptional analysis were predicted as increasing the transcription levels of the genes relating to lipid synthesis and decreasing those relating to lipid degradation to enhance the lipid accumulation, even with the UV-C irradiation as the stress. However, the results of the transcriptomics indicated the promotion of the lipid degradation was dependent on decreasing the transcription levels of *acetyl-CoA carboxylase beta carboxyltransferase*, *acetyl-CoA carboxylase*, *acetyl-CoA biotin carboxyl carrier protein*, *cyclopropane fatty acid synthase* and *3-ketoacyl-ACP-synthase* as shown in Figure 5c and increasing those relating (xi) and (xii) in Figure 5d. Collaborating with the indicated possibility of downregulating the biosynthesis pathway of TAG in Figure 5c, the phenomena of the shifts of the gene transcriptions to generate acetyl-CoA from lipids in Figure 5d (xii) and supporting the depressed gene transcription with other isozyme gene transcription indicated that the cells could respond to the UV-C irradiation by producing acetyl-CoA. As a result, the attempt to supply the acetyl-CoA by degrading the lipids could be connected to the energy production in the TCA cycle, as shown in Figure 5b. Therefore, the cells irradiated by the UV-C as the lethal stress could upregulate the pathway to generate energy rather than to produce lipids. 

## 4. Conclusions

This research was carried out assuming a simpler use of green algae in industry. The convenient use of green algal cells required (1) the maintenance of the intracellular contents in the cells, (2) no environmental contamination even with the direct use of the cells and (3) few changes to the intracellular compositions after the sterilization. Then, *C. reinhardtii*, a model organism of green algae, was irradiated with UV-C, and its cellular responses were investigated. UV-C irradiation, which is generally used for sterilization of microorganisms, could also be lethal to *C. reinhardtii* in 10 min in our irradiation conditions. However, the sterilization effects of the UV-C decreased, even in the same depth, by increasing the cell density, indicating the light-shielding effect of the cells. In this study, the lipids were attracted as the intracellular contents; although the extracted lipids partially showed the change induced by UV-C irradiation, the intracellular lipids showed no change according to the results of no significant difference. On the other hand, as the results of quantitative PCR show, most gene transcription levels relating to the lipid degradation pathway and the TCA cycle to produce energy in the cells were increased by UV-C irradiation. The results provided several interesting findings: although the transcription levels required to obtain energy with lipid degradation increased in response to the UV-C irradiation, the time until death might not be sufficient to degrade the lipids. This was because *C. reinhardtii* showed sufficient responses at the gene transcription level—even though the cells were dying as a result of UV-C irradiation for 10 min—but not at the metabolic flows of lipids. The purpose of this study was to propose a simple use of green algal cells, and this study revealed the properties of retaining the intracellular contents, avoiding microbial contamination in the environments and showing no change in the targeted metabolites, even under the irradiation. This study also showed that CO_2_ as the carbon source could be more effectively used for energy storage rather than energy generation, according to the results of the shifts in transcription levels in glycolysis. 

As a result of transcriptome analysis, the genes to activate the metabolic pathway for lipid degradation were upregulated; however, the time for the response was not enough until cell death, meaning that the lipid contents and composition ratios as metabolites were not affected. Thus, although the UV-C irradiation was a trigger to cause intracellular responses on the gene transcription, there was no significant effect on the lipids. The significant changes of lipid contents and compositions are undesirable for material production, so the UV-C irradiation could be suited for sterilization in lipid production. Herein, it was shown that there would be no problem in recovering the lipids with the cells. This study revealed the cell responses in which the gene transcription level could respond, but the metabolic level could not respond. Those points were progressed points in this study. Therefore, this study could demonstrate significance based on the accumulation of knowledge relating to the response of *C. reinhardtii* to UV-C irradiation. The regulation of the *C. reinhardtii* genes revealed in this study may be relevant to future studies of other stress responses [36]. For example, several selenoproteins related to antioxidant defense activity in animals have been identified in *C. reinhardtii* and may give ideas for future antioxidant and biodefense responses in humans.

## Data Availability

Not applicable.

## Supplementary Materials

The following supporting information can be downloaded at: https://www.mdpi.com/article/10.3390/microorganisms11030633/s1, Supplemental Tables S1~S5 is primer-pair information used in analyses of qPCR. All data were mainly provided by National Center for Biotechnology Information, June 2022.
