# Supplementary material for: Evaluation of Shifts of Gene Transcription Levels of Unicellular Green Alga Chlamydomonas reinhardtii Due to UV-C Irradiation
Source: Microorganisms. 2023 Mar 1;11(3):633. doi: 10.3390/microorganisms11030633 (PMC10059774; doi:10.3390/microorganisms11030633)
Supplement: Supplementary file 1 [file microorganisms-11-00633-s001.zip › microorganisms-2255792-supplementary.pdf]

Supplementary Materials.

Supplemental table S1. List of primer-pair of each enzyme gene performing for quantitative PCR on glycolysis.

Glycolysis	Primer sequences (5'→3')	
ADP-glucose pyrophosphorylase [EC 2.7.7.27] Chromosome:3 (5741796..5746698)	F	tgccagatcatcaacaagga
	R	ccttgatcacgacgacaatg
PGI [EC:5.3.1.9] Chromosome:3 (4379722..4386157)	F	caacaacatcgacgagcact
	R	aggaaggacacgttccacag
FBP [EC:3.1.3.11] Chromosome: 12 (1935474..1938770)	F	gtggtgtccaacgaggtctt
	R	agttgcccgagtaggtctcc
PFK [EC:2.7.1.11] Chromosome:6 (1843743..1848608)	F	gacagcccctttattgtgga
	R	gctctcaaactccaccgtgt
PFK [EC:2.7.1.11] Chromosome:12 (7825525..7831836)	F	gcctggtgaacaagctgact
	R	aagcccctgaatccgtactt
FBA [EC:4.1.2.13] Chromosome:1 (1390088..1396147)	F	gtgtggagaacaccgaggac
	R	cagcgtctcctcaaacatga
FBA [EC:4.1.2.13] Chromosome:2 (2688048..2691909)	F	tatggaagacacggggaatg
	R	tgctaggatgcccttctcag
FBA [EC:4.1.2.13] Chromosome:2 (6427458..6429489)	F	tgaaggagcagaacatcgtg
	R	ccggccttgtagtactcagc
FBA [EC:4.1.2.13] Chromosome:5 (2060640..2063875)	F	cgcaaatagctgcacaactc
	R	caagcgcttcccaatagttc
GAPDH [EC:1.2.1.12] Chromosome:12 (226538..229749)	F	atgactcggtccacaagacc
	R	ctcgggtcgctctcagtaaa
GAPN [EC:1.2.1.9] Chromosome:12 (7427798..7434906)	F	cttgtcctgctttcctcctg
	R	gttgtcaggaagtcgccaat
PGK [EC:2.7.2.3] Chromosome:11 (1638740..1643130)	F	ggtggcatgatcttcacctt
	R	catctcctccagcttcttgg
PGM [EC:5.4.2.12] Chromosome:6 (2875877..2880881)	F	aagcaggagcagtacctgga
	R	cttgtacttgccgctcttga
ENO [EC:4.2.1.11] Chromosome:12 (3521433..3525524)	F	gcctcgtcggagttctacac
	R	gcatctgctccttggtcttc

Supplemental table S2. List of primer-pair of each enzyme gene performing for quantitative PCR on TCA cycle.

TCA cycle	Primer sequences (5'→3')	
PC [EC:6..4.1.1] Chromosome:6 (1351790..1366702)	F	cgaggagtggtccaacatct
	R	gggtcaccttgacgatgtct
CIS [EC:2.3.3.1] Chromosome:3 (1129580..1134612)	F	agtacatcccgccctcctac
	R	ctggtacatgcgcatcatct
CIS [EC:2.3.3.1] Chromosome:12 (3717332..3723387)	F	attggctcggttgagaacat
	R	tcgaagttgcgatacactcg
ACL [EC:2.3.3.8] Chromosome:2 (2024458..2032345)	F	ttcttcggcaagaacgagat
	R	ggaaggaggcgtagttgatg
ACL [EC:2.3.3.8] Chromosome:5 (1845504..1849186)	F	tatgacgctaagcggcttct
	R	aggcatttgtgtttgcagtg
aconitate hydratase [EC:4.2.1.3] Chromosome:1 (6031056..6036984)	F	tgtgctgatcaaggtgaagg
	R	tgagcaggttgttggagatg
isocitrate dehydrogenase [EC:1.1.1.42] Chromosome:1 (6031056..6036984)	F	cctggacctgaagatcgtgt
	R	cacattgtgctccttgatgg
isocitrate dehydrogenase (NAD+) [EC:1.1.1.41] Chromosome:2 (7658830..7663200)	F	caaggagctggacctttacg
	R	ggacggtgaccaggttaatg
isocitrate dehydrogenase (NAD+) [EC:1.1.1.41] Chromosome:17 (3980504..3985464)	F	agaacacaaacacgcagtcg
	R	ctctccctccgtgttctcac
2-oxoglutarate dehydrogenase E1 component [EC:1.2.4.2] Chromosome:12 (6286655..6298508)	F	ccacatcggctacgagtaca
	R	ttctgctgcttggtgaactg
dihydrolipoamide dehydrogenase [EC:1.8.1.4] Chromosome:1 (2774237..2781522)	F	gaccgtgttcacctctgacc
	R	tgtacacgtcgctgaactcc
dihydrolipoamide dehydrogenase [EC:1.8.1.4] Chromosome:1 (232111..238122)	F	agttcaagatgggcaccaag
	R	acgtcgcactccatcttctc
2-oxoglutarate dehydrogenase E2 component [EC:2.3.1.61] Chromosome:7 (4493225..4496996)	F	tcttttcctccacctcctca
	R	ttgacgttgtgcttctccag
2-oxoglutarate dehydrogenase E2 component [EC:2.3.1.61] Chromosome:7 (4536828..4542242)	F	tggagaagcacaacgtcaag
	R	atctcatcgccctcaatcac
succinyl-CoA synthetase alpha subunit [EC:6.2.1.4] Chromosome:3 (6357248..6360946)	F	tggcgtcttttccttactgc
	R	cgcaagtgcagaagactcag
succinate dehydrogenase (ubiquinone) cytochrome b560 subunit [EC:1.3.5.1] Chromosome:1 (3250067..3251938)	F	tcgtacgccatcatctacca
	R	aggaccttgctgctcatctc
succinate dehydrogenase (ubiquinone) iron-sulfur subunit [EC:1.3.5.1] Chromosome:6 (2010871..2014325)	F	ctactggtggaacagcgaca
	R	gcagcggtagagcttgtagg
succinate dehydrogenase (ubiquinone) flavoprotein subunit [EC:1.3.5.1] Chromosome:14 (1657013..1664213)	F	agatcatgaagcccaacacc
	R	tgttctgcatcaccttctgc
fumarate hydratase, class II [EC:4.2.1.2] Chromosome:1 (3224421..3235846)	F	aattcgcaccgttttattgc
	R	tctggttatggtggtgtgga
fumarate hydratase, class I [EC:4.2.1.2] Chromosome:6 (829967..834586)	F	agtacgccaaggaccacatc
	R	actcgtccacgtagctgtcc
malate dehydrogenase [EC:1.1.1.37] Chromosome:2 (7238593..7241569)	F	tgtactcctaccccgtcacc
	R	gtcgatgggtagaccctgaa
malate dehydrogenase [EC:1.1.1.37] Chromosome:3 (6463342..6466758))	F	ctttgtgtcggaggctaagg
	R	gtcatggccttcttctcagc
malate dehydrogenase [EC:1.1.1.37] Chromosome:10 (798235..802160)	F	cactgaaccgcattcaaaag
	R	acacgtaggggctcatcttg
malate dehydrogenase [EC:1.1.1.37] Chromosome:12 (92426..95047)	F	ctacttcgcctccaaggtca
	R	gccttctcgtagtccgacag

Supplemental table S3. List of primer-pair of each enzyme gene performing for quantitative PCR on fatty acid biosynthesis.

Fatty acid biosynthesis		Primer sequences (5'→3')
acetyl-CoA carboxylase beta carboxyltransferase [EC:6.4.1.2] Chromosome:8 (548901..558182)	F	tcttttcgctcatgcagatg
	R	aggtgaggatggcgatgtag
biotin carboxylase [EC:6.4.1.2] Chromosome:8 (548901..558182)	F	tcctccgagtcctacctcaa
	R	gctcagacaggaagccgtag
acetyl-CoA carboxylase [EC:6.4.1.2] Chromosome:12 (4236972..4241816)	F	gaacagaagcatgccaaaca
	R	ccgttgcgtcgtctgtagta
acetyl-CoA biotin carboxyl carrier protein [EC:6.4.1.2] Chromosome:1 (5379991..5382868)	F	ttgtcgggtttgtacgtttg
	R	ctttgtagccaagccctctg
Hypo1 [EC:6.4.1.2] Chromosome:8 (2629396..2653256)	F	gtctggcagctgatcaagag
	R	acaccagctgctccacaac
cyclopropane fatty acid synthase [EC:2.1.1.79] Chromosome:6 (5131766..5139867)	F	ggcctgtacatgagcctgtt
	R	gagccgtagttgagctcctg
3-ketoacyl-ACP-synthase [EC:2.3.1.41]Chromosome:7 (3431377..3438184)	F	tgagccaaccccttttatcag
	R	aaactttgtgagcccagcat
acetyltransferase/acyltransferase [EC:2.3] Chromosome:1 (2837483..2844924)	F	aggtgtgctagtgctgctga
	R	acagcgacagaaggttggag
Hypo2 [EC:2.3.1.15] Chromosome:10 (5641433..5647024)	F	accactacctggcactgagg
	R	caacacatcagccatctcca
lysophosphatidic acid acyltransferase [EC:2.3.1.51] Chromosome:9 (5043586..5049407)	F	aactcagactccgctcctca
	R	ccatcatcgtcacaaacagc
phosphatidate phosphatase [EC:3.1.3.4] Chromosome:5 (664423..668668)	F	cgtgcttgactactggcact
	R	gggtagatgaggcggtacac
diacylglycerol acyl transferase [EC:2.3.1.20] Chromosome:3 (7551370..7554956)	F	cctctttcttagggccgaac
	R	gtgtagatggcgctgacgta

Supplemental table S4. List of primer-pair of each enzyme gene performing for quantitative PCR on fatty acid degradation.

Fatty acid degradation	Primer sequences (5'→3')	
long-chain acyl-CoA synthetase [EC:6.2.1.3] Chromosome:2 (7215449..7219588)	F	gcccctctgattcctacctc
	R	atgtcaccctgccagtatcc
long-chain acyl-CoA synthetase [EC:6.2.1.3] Chromosome:6 (7418052..7425437)	F	agcgcttggtagaggctaca
	R	agcttgtcgccgtaagtgac
long-chain acyl-CoA synthetase [EC:6.2.1.3] Chromosome:12 (2371392..2379925)	F	tacacgtggatgacctacgc
	R	agccactccttgcagttgac
long-chain acyl-CoA synthetase [EC:6.2.1.3] Chromosome:13 (744346..755506)	F	caaggaggacaagctcaagg
	R	aggtcgtcctccacagagaa
acyl-CoA oxidase [EC:1.3.3.6] Chromosome:13 (744346..755506)	F	aggcgaaggtgtaccagatg
	R	cctgcaggattaccttgagc
acyl-CoA oxidase [EC:1.3.3.6] Chromosome:11 (1772882..1781127)	F	ctacgttccgcaatacgaca
	R	agtccgtcaccgaaatgaag
acyl-CoA oxidase [EC:1.3.3.6] Chromosome:16 (4662901..4670018)	F	gacctacgcctaccacttcg
	R	ggatagcacgtggatggtct
acyl-CoA oxidase [EC:1.3.3.6] Chromosome:16 (3731914..3740006)	F	tgattctgaagacgcacacc
	R	gctcctttccaccattcaga
acyl-CoA oxidase [EC:1.3.3.6] Chromosome:16 (165890..174125)	F	caccagcaagatccagaaca
	R	cactggctgaactccatgtc
acyl-CoA dehydrogenase [EC:1.3.8.7] Chromosome:16 (6098446..6108597)	F	actaccgcctcgacaacatc
	R	gtagggcaggcagttgtagg
enoyl-CoA hydratase [EC:4.2.1.17] Chromosome:16 (174286..181836)	F	ttgacatcagccagttccag
	R	catcgttgatggagttgtcg
enoyl-CoA hydratase [EC:1.1.1.35] Chromosome:16 (174286..181836)	F	ttgacatcagccagttccag
	R	catcgttgatggagttgtcg
enoyl-CoA hydratase [EC:1.1.1.211] Chromosome:16 (174286..181836)	F	ttgacatcagccagttccag
	R	catcgttgatggagttgtcg
acetyl-CoA acyltransferase [EC:2.3.1.16] Chromosome:17 (3368170..3371811)	F	agatcaccatcagcgaggac
	R	tgccgttcttcttgaacaca
acetyl-CoA C-acetyltransferase [EC:2.3.1.9] Chromosome:2 (7215449..7219588)	F	gcttcgaaagcatgagcaac
	R	tcccatagtccgtccttcac

Supplemental table S5. List of primer-pair of each enzyme gene performing for quantitative PCR on housekeeping.

Housekeeping gene	Primer sequences (5'→3')	
ATPS (ATPase beta chain)	F	gaccctgctcctgctacaac
	R	ggtcaaccgcagggtaaata
ARPC3 (actin-related protein Arp2/3 complex, subunit ARPC3-gene)	F	caggccaaattccctgttta
	R	caccctctctccacttacgc
ARP3 (actin-related protein Arp2/3 complex, subunit Arp3-gene)	F	cgggagcagtaccaggagta
	R	tgcgtctcggaagacgtagt
ARPC4 (actin-related protein Arp2/3 complex, subunit ARPC4-gene)	F	ttagggataacccggagctt
	R	gctgacgcgtacagagttga
ARP2 (actin-related protein Arp2/3 complex, subunit Arp2-gene)	F	agcaccagcaactgaaggac
	R	atctcctcccatgactgcac
